# Metabolic and proteomic indications of diabetes progression in human aqueous humor

**DOI:** 10.1371/journal.pone.0280491

**Published:** 2023-01-18

**Authors:** Christopher R. Fortenbach, Jessica M. Skeie, Kristina M. Sevcik, A. Tim Johnson, Thomas A. Oetting, Jaclyn M. Haugsdal, Christopher S. Sales, Darryl Y. Nishimura, Eric B. Taylor, Gregory A. Schmidt, Mark A. Greiner

**Affiliations:** 1 Department of Ophthalmology and Visual Sciences, University of Iowa, Iowa City, IA, United States of America; 2 Iowa Lions Eye Bank, Coralville, IA, United States of America; 3 Carver College of Medicine, University of Iowa, Iowa City, IA, United States of America; 4 Department of Biochemistry, Fraternal Order of the Eagles Diabetes Research Center, Abboud Cardiovascular Research Center, Holden Comprehensive Cancer Center, and Pappajohn Biomedical Institute, University of Iowa Carver College of Medicine, Iowa City, IA, United States of America; Pacific Northwest National Laboratory, UNITED STATES

## Abstract

Diabetes mellitus is a multiorgan systemic disease impacting numerous ocular structures that results in significant ocular morbidity and often results in more frequent corneal and glaucoma surgeries for affected individuals. We hypothesize that the systemic metabolic and proteomic derangement observed in the progression of diabetes influences the composition of the aqueous humor (AH), which ultimately impacts the anterior segment health of the eye. To identify changes associated with diabetes progression, we mapped the metabolite profile and proteome of AH samples from patients with varying severities of type II diabetes (T2DM). Patients were classified as nondiabetic (ND or control), non-insulin-dependent diabetic without advanced features of disease (NAD-ni), insulin-dependent diabetic without advanced features (NAD-i), or diabetic with advanced features (AD). AH samples collected from the anterior chamber during elective ophthalmic surgery were evaluated for metabolite and protein expression changes associated with diabetic severity via gas chromatography/mass spectrometry and ultra-high performance liquid chromatography tandem mass spectrometry, respectively. Metabolic and proteomic pathway analyses were conducted utilizing MetaboAnalyst 4.0 and Ingenuity Pathway Analysis. A total of 14 control, 12 NAD-ni, 4 NAD-I, and 14 AD samples were included for analysis. Elevated levels of several branched amino acids (e.g., valine, leucine, isoleucine), and lipid metabolites (e.g., palmitate) were found only with increasing diabetic severity (i.e., the AD group). Similar proteomic trends were noted in amino acid and fatty acid metabolism and the unfolded protein/stress response. These results represent the first report of both metabolomic and proteomic evaluation of aqueous humor. Diabetes results in metabolic and proteomic perturbations detectable in the AH, and unique changes become manifest as T2DM severity worsens. Changes in AH composition may serve as an indicator of disease severity, risk assessment of anterior segment cells and structures, and potential future therapies.

## Introduction

Diabetes is a chronic, metabolic disease characterized by elevated levels of blood glucose leading to multiorgan system damage. Its global prevalence has nearly doubled over the past decade, with over a half billion cases projected by 2030 [1]. Of the many possible sequalae of diabetes, approximately one in ten will suffer vision-threatening eye disease including diabetic retinopathy, cataract, and other anterior segment diseases [2]. In particular, the impact of corneal endothelial cell function in the setting of diabetes has been an area of extensive study. Diabetic patients have impaired corneal endothelial cell function [3–7], decreased corneal endothelial cell density [7–9], and structural changes [10, 11], all of which likely contribute to the poorer surgical outcomes that have been observed in endothelial keratoplasty (e.g., Descemet Membrane Endothelial Keratoplasty or DMEK) [12] and cataract surgery [7, 9]. Despite surgical improvements which can help mitigate some of these differences, the underlying causes of anterior segment dysfunction in diabetic patients remain unknown.

Similar to the pathologic changes observed in diabetic serum, perturbations in the metabolomic and proteomic composition of aqueous humor may contribute to the dysfunction of adjacent anterior segment structures in diabetic patients [13, 14]. Early studies on advanced diabetics have shown several concordant changes including decreased levels of antioxidants (e.g., methyltetrahydrofolic acid, taurine, niacinamide, and xanthine), changes in amino acid concentrations (e.g., decreased succinate), and an increase in glycosolated amino acids [15–17]. Extensive changes in protein expression including nutrient transport, angiogenesis, antioxidants and the complement cascade have also been observed [16, 18]. Previous studies have focused on advanced diabetic disease rather than stratifying by diabetes severity and no ophthalmic study to date has concurrently assessed the metabolomic and proteomic changes of diabetes.

We conducted metabolomic and proteomic analyses of aqueous humor sampled from the anterior chamber during elective ophthalmic surgery from patients with and without type II diabetes mellitus (T2DM). T2DM patients were stratified by diabetes severity and AH samples underwent metabolomic and proteomic analysis.

## Materials and methods

### Ethical approval

This study was approved by the University of Iow’’s Institutional Review Board (IRB #201603746) and adhered to the tenants of the Declaration of Helsinki.

### Aqueous humor collection and grouping

Patients with and without documented T2DM undergoing anterior segment surgery at the University of Iowa Hospitals & Clinics were enrolled in the study beginning in 2016. Only adults (> 18 years of age) were enrolled and written informed consent was obtained. Following a comprehensive review of their medical history, diabetic severity was classified based on the modified Diabetic Complications Severity Index (DCSI; [19, 20]): nondiabetic controls (ND), nonadvanced diabetic without insulin dependence (NAD-ni), nonadvanced diabetic with insulin dependence (NAD-i), and advanced diabetic (AD, defined as having end-organ complications secondary to diabetes with insulin dependence). Myocardial infarction was not considered a complication specific to diabetes for any of these groups due to its high incidence in the US population. Patients with a history of prior incisional surgery or current steroid use were excluded.

Aqueous humor samples were collected in the operating room following the first corneal incision during anterior segment surgery. Volumes between 50-100 μL were obtained by manual aspiration using a 30g cannula from the anterior chamber. Samples were frozen immediately and transferred subsequently to the Iowa Lions Eye Bank and stored at -80° C.

### Metabolite analysis

Metabolomic analysis via gas chromatography/mass spectrometry (GC-MS) was performed at the Fraternal Order of Eagles Diabetes Research Center Metabolomics Core in the Carver College of Medicine. For GC-MS metabolite profiling, samples were extracted with a methanol-acetonitrile-sample mix in a 2:2:1 ratio followed by conversion to a trimethylsilyl derivative in order to make the metabolites of interest amenable to GC-MS [21].

Metabolites were separated by gas chromatography and detected using either a single quadrupole mass spectrometer (MS) or a Q-Exactive (QE) MS (ThermoFisher). Data were collected in either full mass range (50-700 Da) or by single ion monitoring (SIM), which focused on selected ions of interest only. A set of 9 isotopically labeled internal standards were added prior to sample extraction providing for correction of extraction, derivatization and/or loading effects. The standards are as follows: D4-Succinate (Sigma-Aldrich, 492248-5G); D8-Valine (Cambridge Isotope Laboratories; DLM-488-0.5); D4-Citrate (Cambridge Isotope Laboratories, DLM-3487-PK); 13C5-Glutamine (Cambridge Isotope Laboratories, CLM-1822-H-PK); 13C5-Glutamate (Cambridge Isotope Laboratories, CLM-1800-H-PK); 13C6-Lysine (Cambridge Isotope Laboratories, CLM-2247-H-PK); 13C5-Methionine (Cambridge Isotope Laboratories, CLM-893-H-PK); 13C3-Serine (Cambridge Isotope Laboratories, CLM-1574-H-PK)13C11-Tryptophan (Cambridge Isotope Laboratories, CLM-4290-H-PK). Pooled Quality Control (QC) samples were injected in duplicate at the beginning and end of each run, as well as after every 10 runs to correct for instrument drift over time using local regression analysis by NOREVA software [22].

Identification of metabolites in a sample were based on comparison to an in-house mass spectrum library of authenticated standards and their retention times using Tracefinder 4.1 (Thermo) software [23].

### Protein analysis

Aqueous humor samples were prepared and quantified using ultra-high performance liquid chromatography tandem mass spectrometry (UHPLC-MS/MS) as previously described [24, 25]. Briefly, digested peptides were collected by centrifugation and approximately 20 μg of peptides were desalted using reversed phase stop-and-go extraction (STAGE) tips [26], eluted with 80% acetonitrile and 5% ammonium hydroxide, and lyophilized in a SpeedVac (Thermo Fisher Scientific; Waltham, MA) for 1 hour. Liquid chromatography (LC) was performed on an Easy-nLC 1000 UHPLC system (Thermo Fisher Scientific) and then LC was interfaced to a quadrupole-Orbitrap mass spectrometer (Q-Exactive; Thermo Fisher Scientific). The mass spectrometer acquired tandem mass spectra from the top 20 ions in the full scan from 400 – 1200 m/z while dynamic exclusion was set to 15 seconds, isolation width to 1.6 Dalton, full MS resolution to 70,000, and MS/MS resolution to 17,500; singly-charged ions were excluded. Normalized collision energy was set to 25 eV, automatic gain control to 2e5, max fill MS to 20 milliseconds, max fill MS/MS to 60 milliseconds, and underfill ratio to 0.1%. RAW data files were converted to mzML format using msconvert [27], then MGF files were generated from mzML using the Peak Picker HiRes tool from the OpenMS framework [28]. MGF files were searched using up-to-date protein sequence libraries available from UniProtKB, X!Tandem [29], and OMSSA [30].

### Bioinformatics and statistical analysis

A systems biology approach was implemented using bioinformatics analyses to determine significant protein expression (Partek Geonomics Suite 6.6; Partek Inc., St. Louis, MO), and pathway representation (Ingenuity Pathway Analysis, IPA; Qiagen, Germantown, MD). Metabolite intensities were scaled to logarithmic base 2, and statistically significant metabolites (analysis of variance [ANOVA], p < 0.05) were used to determine canonical pathway representation. Similarly, protein intensities were scaled to logarithmic base 10, and statistically significant proteins (analysis of variance [ANOVA], p < 0.05) were used to determine canonical pathway representation. Pathway significance was determined by the number of proteins in the dataset in common with known proteins in a single pathway. Proteins and metabolites were both analyzed for significant pathways altered in diabetes progression, as well as compared to each other to determine significant pathways affected by both metabolite and proteomic alterations. In addition, GO categorization for statistically significant proteins (**S1 Fig**) were determined using PANTHER classification system (version 17.0) and protein function for individual proteins was referenced from both the Genecards and Uniprot databases (**Table 4**).

## Results

### Patient demographics

Aqueous humor samples were collected from a total of 44 eyes from 41 patients (14 controls, 12 NAD-ni, 4 NAD-i, and 14 AD; **Table 1**). The mean age of the diabetic patients was 68.0 years +/- 2.0 years with no statistically significant difference in age between the control and diabetic cohorts (p > 0.47). The mean BMI among the diabetic patients was 36.5 +/- 3.7, 37.7 +/- 0.84, and 36.6 +/- 1.8 in the NAD-ni, NAD-i, and AD subgroups, respectively. There was no significant difference between BMI among the diabetes subgroups (p > 0.74). The mean duration of diabetes was 12.1 +/- 2.6, 12.2 +/- 4.5, and 22.8 +/- 2.3 years for the NAD-ni, NAD-i, and AD groups, respectively. AD patients had a significantly longer duration of diabetes compared to the other groups (p < 0.043).

**Table 1:** Patient demographics. Demographic, ocular, and diabetic history for each study participant. Each line corresponds to a single patient and where more than one sample number is listed, the second samples was collected from the fellow eye. Proteomic analysis was performed on all samples denoted with an asterisk.

Sample	Diagnosis	Age	Sex	Race	Ocular diagnoses	Ocular Medications	DM Duration (years)	DM Medications
1	NAD-ni	75	M	Caucasian	None		19	Metformin
2	NAD-ni	73	F	Caucasian	None		5	Metformin
3	NAD-ni	51	M	Caucasian	None		0.5	
4*	NAD-ni	78	F	Caucasian	Dry eye, PVD, drusen		Unknown	
5	NAD-ni	84	M	Caucasian	Exudative AMD	Anti-VEGF	Unknown	Glipizide
6	NAD-ni	74	M	Caucasian	None		8	Metformin, glimepiride
7	NAD-ni	66	M	Caucasian	None		Unknown	
8*	NAD-ni	76	F	Caucasian	History of giant papillary conjunctivitis, MGD		21	Metformin, glimepiride
9*	NAD-ni	42	M	Caucasian	Idiopathic intracranial hypertension		8	Metformin
10	NAD-ni	69	M	Caucasian	Epiretinal membrane, PVD		21	Metformin, glimepiride
11	NAD-ni	75	M	Hispanic/Latino	None		Unknown	Metformin
12*	NAD-ni	68	F	Caucasian	Exudative AMD	Anti-VEGF	14	Metformin
13*, 14*	NAD-i	76	F	Caucasian	Non-exudative AMD, PVD		20	Insulin, metformin
15*	NAD-i	65	F	African American	Glaucoma suspect		5	Insulin
16	NAD-i	64	F	African American	Dry eye		4	Insulin, metformin
17*	AD	81	M	Caucasian	Moderate NPDR, pterygium, PVD		35	Insulin
18*	AD	66	M	Caucasian	PDR, DME, ERM		26	Insulin
19	AD	57	F	Caucasian	PDR, DME, VH, band keratopathy, drusen		26	Insulin
20*, 21*	AD	65	M	Hispanic/Latino	NPDR, pterygium, MGD		31	Insulin, metformin
22*	AD	69	F	Caucasian	None		7	Metformin
23	AD	70	M	Caucasian	None		11	Insulin
24	AD	74	F	Caucasian	Chalazia, asteroid hyalosis, MGD		22	Insulin
25	AD	66	M	African American	None		15	Insulin, metformin
26, 27	AD	61	M	Caucasian	None		20	
28	AD	72	F	Caucasian	None		Unknown	
29	AD	55	F	Caucasian	NPDR		28	Insulin
30	AD	59	M	Caucasian	PDR, macular edema	Anti-VEGF	24	Insulin
31*	Control	77	F	Caucasian	Dry eye, MGD	Topical cyclosporine	-	-
32	Control	37	M	African American			-	-
33*	Control	72	F	Caucasian			-	-
34*	Control	82	M	Caucasian	MGD	Doxycycline	-	-
35	Control	76	F	Caucasian			-	-
36	Control	28	M	African American	Chronic allergic conjunctivitis	Ketotifen	-	-
37	Control	79	F	Caucasian	Dry AMD, MGD	Topical cyclosporine	-	-
38	Control	78	F	Declined	ABMD		-	-
39	Control	65	M	Caucasian			-	-
40	Control	76	M	Caucasian	Dry AMD, ABMD		-	-
41*	Control	80	F	Caucasian	Amblyopia, exotropia		-	-
42	Control	89	M	African American			-	-
43	Control	67	F	Caucasian	MGD, Salzmann nodule degeneration	Ketotifen	-	-
44	Control	53	F	African American	High myopia, MGD		-	-

ABMD: anterior basement membrane dystrophy; AMD: age-related macular degeneration; MGD: meibomian gland dysfunction; NPDR: non-proliferative diabetic retinopathy; PDR: proliferative diabetic retinopathy; PVD: posterior vitreous detachment; VH: vitreous hemorrhage

### Metabolite changes associated with diabetes progression

**Fig 1:**
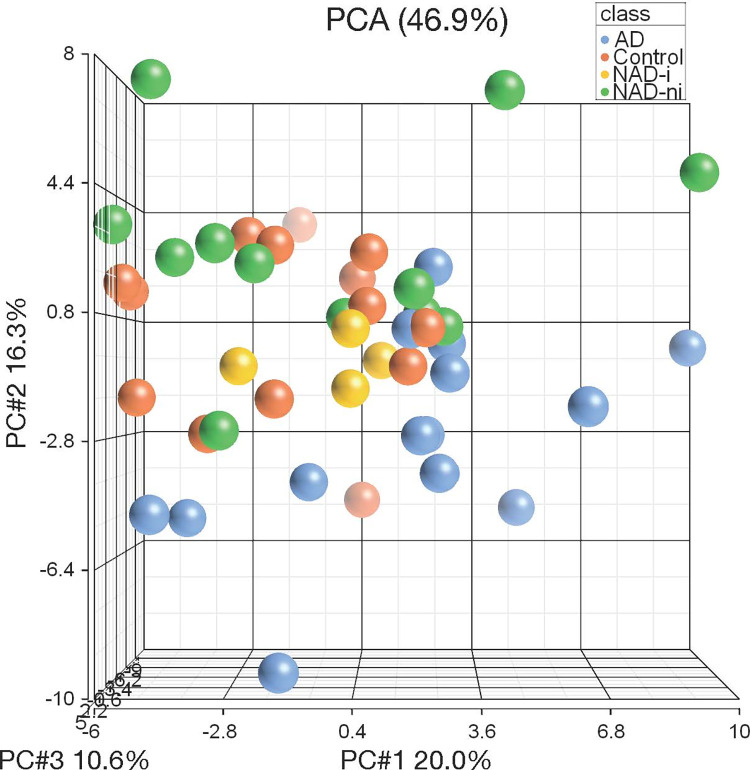
Metabolomic principal component analysis. Principal component analysis of the GC-MS metabolomic data from aqueous humor samples from control (orange), NAD-ni (green), NAD-i (yellow), and AD (blue) patients. Considerable overlap was observed between the NAD-ni and control patients, whereas the NAD-i patients grouped between the NAD-ni and AD groups.

Aqueous humor samples were subject to GC-MS to evaluate for metabolic changes associated with diabetes progression. A total of 70 metabolites were evaluated across all samples and compared to controls. Principal component analysis showed significant overlap between the control and NAD-ni patients, whereas the NAD-i patients grouped between the NAD-ni and AD groups (**Fig 1**).

**Fig 2:**
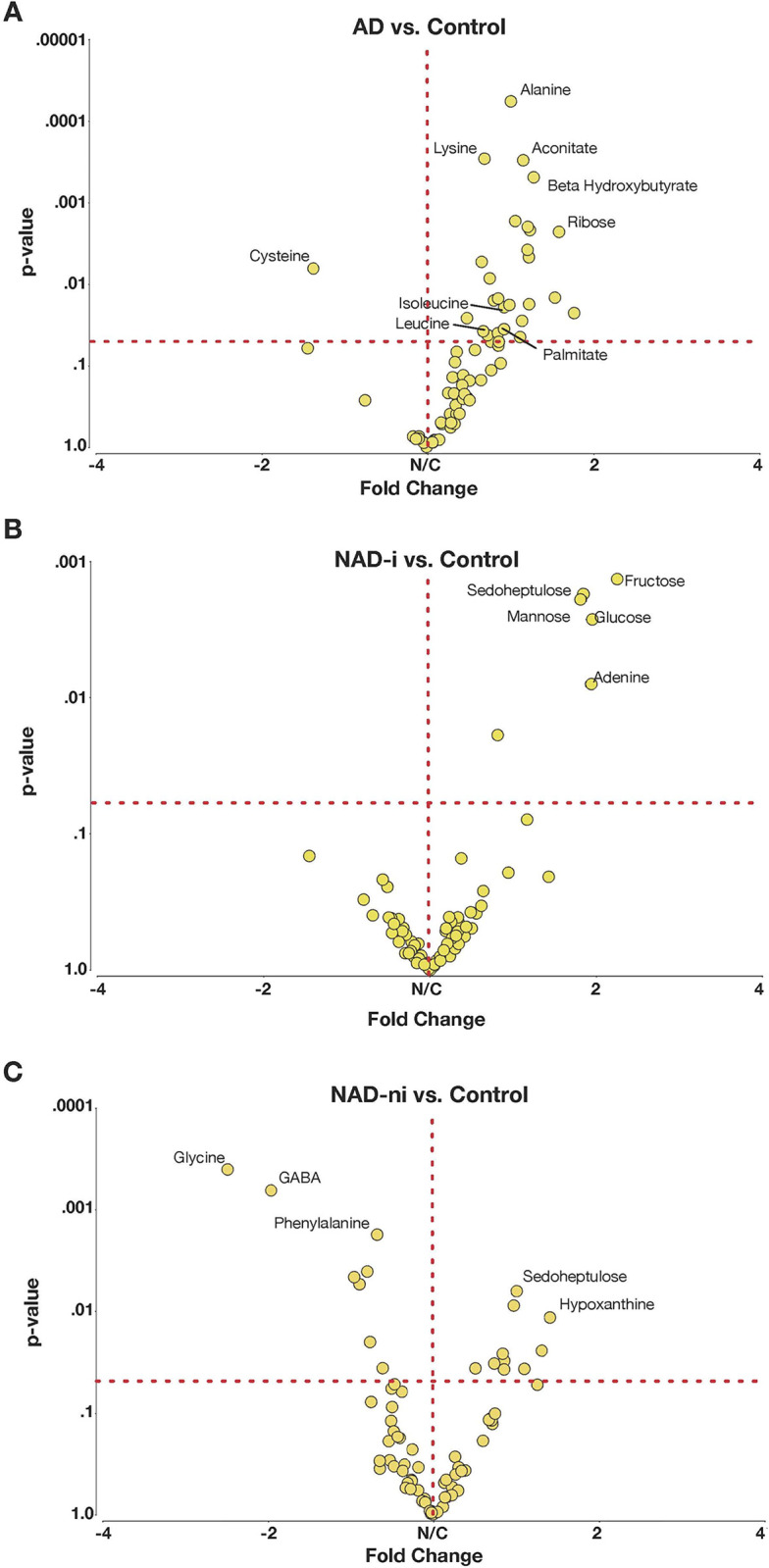
Changes in metabolome associated with diabetes severity. Volcano plots showing the relative fold changes in metabolite concentration between diabetic subgroups and controls. The dashed line is drawn at p = 0.05. Significant changes are noted in carbohydrate, amino acid, and lipid metabolsim across diabetic severities.

Significant metabolic differences were detected across diabetic severities. Multiple carbohydrates and glycolytic intermediates were present at elevated levels including glucose, fructose, ribose, and mannose (**Table 2; S1 Table**). Conversely, multiple amino acids were found at decreased levels including asparagine, cysteine, threonine, tryptophan, and tyrosine. Interestingly, these differences were often only detected among NAD-ni (e.g., aromatic amino acids) or only among the AD group (e.g., cysteine). The branched amino acids including isoleucine and valine were detected at elevated levels more frequently with progressive diabetes severity. Components of lipid metabolism were also altered among diabetic patients with increased levels of beta-hydroxybutyrate observed across multiple severities, whereas palmitate was elevated only among the most severe diabetic subgroup. These findings are summarized in the volcano plots in **Fig 2**.

**Table 2:** Selected metabolic changes associated with diabetes progression. Fold-changes in aqueous humor metabolite concentration among diabetic patients and controls. Statistically significant values for individual comparisons (p < 0.05) are highlighted in yellow. The final column represents the p-value based on a 4-way analysis. A complete list of metabolites can be found in **S1 Table**.

Metabolite	NAD-ni v ND	NAD-I v ND	AD v ND	NAD-i v NAD-ni	AD v NAD-ni	AD v NAD-i	p-value
Alanine	1.19	1.33	1.41	1.12	1.19	1.07	0.00062
Asparagine	-1.24	1.03	1.16	1.27	1.44	1.13	0.0081
Aspartate	-1.18	-1.01	1.06	1.16	1.25	1.07	0.070
Beta Hydroxybutyrate	1.40	1.01	1.56	-1.38	1.11	1.54	0.0019
Cysteine	-1.20	1.16	-1.61	1.39	-1.34	-1.87	0.020
Fructose	1.28	2.18	1.25	1.71	-1.02	-1.75	0.013
Glucose	1.27	1.97	1.52	1.55	1.20	-1.29	0.0058
Isoleucine	-1.05	1.02	1.38	1.07	1.44	1.35	0.039
Leucine	-1.04	1.04	1.26	1.08	1.31	1.21	0.087
Mannose	1.34	1.87	1.52	1.40	1.13	-1.24	0.0028
Palmitate	1.29	1.21	1.37	-1.06	1.06	1.13	0.17
Phenylalanine	-1.27	-1.05	1.11	1.21	1.41	1.16	0.00027
Ribose	1.46	1.19	1.73	-1.23	1.18	1.45	0.017
Threonine	-1.37	-1.08	1.13	1.26	1.54	1.22	0.0020
Tryptophan	-1.32	-1.04	1.06	1.27	1.40	1.10	0.0045
Tyrosine	-1.31	-1.12	1.10	1.17	1.43	1.23	0.017
Valine	-1.04	1.12	1.29	1.17	1.35	1.15	0.016

Given the differences in systemic diabetes medications within each diabetic cohort, metabolite comparisons were also made with and without metformin, insulin, and glimepiride. While only trivial differences were observed due to glimepiride (only urea reaching statistical significance), metformin and insulin showed differing levels of several carbohydrates and amino acids in the NAD-ni and AD cohorts, respectively. Comparisons were limited to the diabetic severity in which the greatest number of patients were taking the medication in question. A complete list of statistically significant metabolites can be found in **S2 Table**.

### Metabolic pathways impacted with diabetes progression

A molecular pathway analysis of the statistically significant metabolites among diabetic patients identified multiple impacted pathways (**Table 3**). Consistent with the differences noted among individual metabolites, multiple amino acid synthesis and degradation pathways were affected in addition to carbohydrate metabolism, glutathione metabolism, and NAD biosynthesis. A complete list of affected pathways is available in **S3 Table**.

**Table 3:** Top 12 metabolic pathways impacted by diabetes progression (4-way analysis). Pathways are ranked by p-value and the ratio represents the number of metabolites (seen in the next column) impacted relative to the total number of metabolites involved in the pathway.

Ingenuity Canonical Pathways	-log(p-value)	Ratio	Molecules
tRNA Charging	14.1	0.302	glycine, L-alanine, L-asparagine, L-cysteine, L-glutamine, L-isoleucine, L-lysine, L-methionine, L-phenylalanine, L-threonine, L-tryptophan, L-tyrosine, L-valine
Glycine Biosynthesis III	4.81	0.75	glycine, L-alanine, pyruvic acid
Glycine Betaine Degradation	4.43	0.308	glycine, L-homocysteine, L-methionine, pyruvic acid
Alanine Biosynthesis III	3.59	1	L-alanine, L-cysteine
Cysteine Biosynthesis III (mammalia)	3	0.231	L-cysteine, L-homocysteine, L-methionine
Thio-molybdenum Cofactor Biosynthesis	2.82	0.5	L-alanine, L-cysteine
Alanine Degradation III	2.82	0.5	L-alanine, pyruvic acid
Alanine Biosynthesis II	2.82	0.5	L-alanine, pyruvic acid
L-cysteine Degradation II	2.82	0.5	L-cysteine, pyruvic acid
Tyrosine Biosynthesis IV	2.82	0.5	L-phenylalanine, L-tyrosine
Superpathway of Methionine Degradation	2.72	0.118	L-cysteine, L-homocysteine, L-methionine, pyruvic acid
Citrulline Biosynthesis	2.57	0.167	citrulline, L-glutamine, L-ornithine

### Changes in protein expression with diabetes progression

A random subset of samples (four each from NAD-ni and AD; three from NAD-i) was subjected to proteomic evaluation, which identified 56,400,212 residues corresponding to 188,558 entries in Uniprot (9606–- Homo sapiens taxa). A total of 1,114 protein isoforms were found to statistically differ across diabetic severities (e.g., p < 0.05 on a 4-way ANOVA). The full proteomic dataset is available in the supplemental data (**S4 Table**). Across all comparisons (e.g., NAD-ni v NAD-I; NAD-ni v AD, etc.), expression of 408 proteins increased on average while 549 decreased (**S5 Table**). Among those proteins with changes in expression, they broadly had roles including not limited to metabolism, immunity, transcription, translation, and cellular structure (summarized in **Table 4** and **S1 Fig**).

**Table 4:** Top 15 proteins impacted by diabetes progression (4-way analysis). Fold-changes in aqueous humor peptide count among diabetic patients and controls. A representative example of each protein function obtained from the Genecards and Uniprot databases is provided. Statistically significant values for individual comparisons (p < 0.05) are highlighted in yellow. The final column represents the p-value based on ANOVA analysis of all 4 groups. A complete list and individual group contrast p-values can be found in **S4 Table**.

UniProt ID	Function	NAD-ni v ND	NAD-i v ND	AD v ND	NAD-i v NAD-ni	AD v NAD-ni	AD v NAD-i	p-value
B4DWA1	Plasma membrane organization	-4.08	1.05	-1.52	4.30	2.68	-1.60	0.00017
P62942	Protein folding	-1.73	1.19	1.40	2.06	2.41	1.17	0.00061
A0A0A0MT76	Adaptive immune response	1.04	-2.60	-2.49	-2.70	-2.59	1.04	0.00063
O95568	Regulation of translation	2.09	-1.67	-2.45	-3.50	-5.13	-1.46	0.00064
F8W6I7	mRNA processing	1.15	-1.13	5.27	-1.31	4.57	5.98	0.00068
P07108	Fatty acid metabolism	-12.20	-1.31	-4.32	9.28	2.83	-3.28	0.00104
H0YH80	RNA binding protein	-1.03	1.19	6.86	1.22	7.05	5.76	0.00106
Q5VZR0	Protein homodimerization activity	-3.84	1.56	-1.77	6.01	2.17	-2.77	0.00128
Q9UEK9	Scaffold protein binding	-2.65	-1.18	-1.49	2.26	1.79	-1.26	0.00149
K7EJ44	Actin cytoskeleton organization	-2.14	1.08	-4.19	2.32	-1.96	-4.54	0.00203
P02663	Protein homodimerization activity	10.97	3.78	4.98	-2.90	-2.20	1.32	0.00220
P05109	Apoptotic process	-2.55	1.23	-1.88	3.14	1.35	-2.32	0.00221
Q65ZC8	Adaptive immune response	6.38	1.54	1.52	-4.14	-4.20	-1.01	0.00235
E7EPL9	Fatty acid beta-oxidation	-15.18	1.18	-1.31	17.92	11.58	-1.55	0.00257
Q05CV3	Complement activation	-1.25	-1.57	-9.66	-1.26	-7.71	-6.13	0.00262

A molecular pathway analysis of the statistically significant proteins differentially expressed by disease severity identified several groups of functionally related proteins. The top global pathways related to lipid metabolism, amino acid metabolism, and inflammation (**Table 5**). A complete listing of differentially expressed proteins by molecular pathway can be found in **S6 Table**. Among those metabolomic and proteomic pathways impacted by diabetes progression, both glycolysis and gluconeogenesis were impacted. A consolidated table showing the common metabolomic and proteomic pathways impacted by diabetes can be found in **S7 Table**.

**Table 5:** Top 12 proteomic pathways impacted by diabetes progression. Pathways are ranked by p-value and the ratio represents the number of peptides (seen in the next column) impacted relative to the total number of peptides involved in the pathway.

Ingenuity Canonical Pathways	-log(p-value)	Ratio	Molecules
Complement System	6.9	0.162	C1QBP, C3, C8B, CFD, CFH, ITGAM
EIF2 Signaling	4.82	0.0402	HNRNPA1, RPL18, RPL35, RPL6, RPS27, RPS3, RPS3A, RPSA, WARS1
Tryptophan Degradation X (Mammalian, via Tryptamine)	4.59	0.148	AKR1B10, ALDH1A1, ALDH2, ALDH3A1
Actin Cytoskeleton Signaling	4.51	0.0367	ACTR3, ARPC1B, ARPC2, BAIAP2, F2, FN1, ITGAM, MYH14, PFN1
Clathrin-mediated Endocytosis Signaling	4.43	0.0415	ACTR3, ARPC1B, ARPC2, DNM3, F2, RAB4A, RAB4B, S100A8
Role of IL-17A in Psoriasis	4.05	0.214	S100A7, S100A8, S100A9
Regulation of Actin-based Motility by Rho	3.97	0.0517	ACTR3, ARPC1B, ARPC2, BAIAP2, ITGAM, PFN1
Telomere Extension by Telomerase	3.95	0.2	HNRNPA1, HNRNPA2B1, XRCC5
RAC Signaling	3.56	0.0435	ACTR3, ARPC1B, ARPC2, BAIAP2, CYBB, ITGAM
Histamine Degradation	3.5	0.143	ALDH1A1, ALDH2, ALDH3A1
Fatty Acid α-oxidation	3.5	0.143	ALDH1A1, ALDH2, ALDH3A1
Actin Nucleation by ARP-WASP Complex	3.47	0.0538	ACTR3, ARPC1B, ARPC2, BAIAP2, ITGAM

## Discussion

In the present study, we show that the AH metabolome and proteome are altered by diabetes. Our results demonstrate that changes in AH composition were dependent on disease severity compared to controls. To our knowledge, this is the first published study to report concurrent metabolomic and proteomic data from human aqueous humor in patients with diabetes. This complete set of data will support future efforts to determine accurate biomarkers of diabetes progression and may provide potential targets for future therapeutics to prevent vision loss.

In a systemic disease often characterized by hyperglycemia, it is unsurprising that glucose and other glycolytic intermediates were elevated with greater frequency among more severe diabetic patients. This aligned with previous studies that have shown impaired glycolysis in diabetic samples of aqueous humor [31] and the corneal endothelium [25]. Elevated glucose has been implicated in diabetic damage via downstream activation of protein kinase C, ultimately resulting in abnormalities of blood flow, decreased nitric oxide production, and induced VEGF expression [32]. It has also been postulated that excess glucose resulting in advanced glycation end products is a mechanism of ocular damage [20], and elevated levels of glycosylated amino acids have been found in aqueous humor of diabetics [15]. Advanced glycation end products are understood to cause cellular damage by altering the function of modified proteins and by producing reactive oxygen species [32]. This may contribute to the observed oxidative stress in the anterior chamber and provide an explanation for the role of glycolytic changes in altered anterior segment health.

In addition to glycolytic intermediates, elevated levels of branched chain amino acids have been associated with insulin resistance and diabetes related disease. While previous studies have shown elevated levels of branched amino acids in the vitreous of patients with diabetic retinopathy [31], here we show the branched amino acids were produced at higher levels only among the most severe diabetics. That this was only observed among individuals with advanced diabetic disease may help explain why prior evaluations of diabetic aqueous humor failed to observe such an increase [15]. The presence of elevated levels of branched chain amino acids is thought to result in glutamate excitotoxicity by competitively inhibiting branch chain aminotransferase and decreasing glutamate transamination, as shown in diabetic retinas [33] and possibly resulting in increased oxidative stress [34]. While this process has been implicated in the progression of diabetic retinopathy, it remains to be determined if it has a role in changes of the anterior segment related to diabetes.

Lipid metabolism was also impacted with elevated palmitate levels observed in the advanced diabetic patients. Excess fatty acids, including palmitate, have been well-recognized to induce reactive oxygen species in a variety of tissues, including cardiac, vascular smooth muscle, hepatic, and pancreatic beta cells [35]. Furthermore, palmitate has been shown to play a role in oxidative stress and subsequent mitochondrial dysfunction, resulting in damage to corneal endothelial cells [36]. While the observed elevation in palmitate may play a role in impacting anterior segment health, it should also be noted that there was less than a 2-fold increase in palmitate overall in the diabetic patients, which may not be sufficient to induce these changes.

Proteomic analyses of the aqueous humor have been reported across a multitude of ocular disease including diabetes. Most of these studies have focused on proliferative diabetic retinopathy [18] with considerably less known about the changes that occur due to more mild disease or with progression. To the best of the authors’ knowledge, this study represents the first evaluation of proteomic changes associated with stratified severities of diabetes relative to controls.

Multiple proteins associated with amino acid and fatty acid metabolism were impacted by diabetic severity (e.g., ALDH1A1, ALDH2, ALDH3A1). ALDH3A1, in addition to being involved in amino acid/fatty acid metabolism, is a regulator of oxidative stress-associated cellular processes [37, 38]. In a recent proteomics study by Chen and colleagues [39], it was found to be significantly upregulated in patients with diabetic retinopathy that underwent anti-VEGF treatments. Interestedly, in addition to be upregulated in the AD group, insulin-dependent non-advanced diabetic patients (NAD-i) also showed significant upregulation despite the absence of proliferative diabetic retinopathy. Given its role in angiogenesis [40], ALDH3A1 could aid in guiding treatment or serve as a biomarker of neovascularization prior to presence of clinical proliferative disease.

Eukaryotic initiation factor 2 (EIF2) complex signaling participates in the unfolded protein/stress response and ultimately cell cycle arrest, and/or cellular apoptosis [41]. Mammalian target of rapamycin (mTOR) similarly play a role in cell survival, autophagy, and insulin signaling [42, 43]. Diabetic severity impacted both of these signaling pathways. While we have previously reported similar results in the proteomic analysis of endothelium-Descemet membrane tissue, similar findings have not been reported in aqueous humor. Given that the two complexes work together to maintain cell growth and metabolism balance [43], perturbations associated with diabetes have the potential to contribute to the poorer cataract and corneal transplant outcomes in diabetic patients [7, 9, 12].

This study provides a unique dataset in that it is the first to include both metabolomic and proteomic analysis of aqueous humor from the same patients across a range of T2DM disease severities. It can be used as a resource for further investigations related to the biochemical impacts of diabetes progression on the anterior segment. The bioinformatics results strongly support previous findings relating diabetes to oxidative damage and are similar to previous reports in the systemic literature. A limitation of this study is its relatively small sample size, the limited number of metabolites evaluated, and the presence of confounding variables among the patients, including comorbid conditions and medications.

The impact of systemic diabetic medications on ocular metabolism remains relatively unknown. In one of the only studies to report on the topic, metformin has been detected in the aqueous humor, but no significant metabolic differences were detected [15]. The present study, which was larger and segregated subjects by diabetic severity, noted an increase in glycine, malate, and phenylalanine, which are also increased in the plasma of diabetic patients on metformin [44–46]. While the present study was not specifically designed to evaluate the impacts of systemic diabetic medications, the concordant results with the systemic literature may indicate that these medications also have an impact on intraocular metabolism and may therefore play a role in the development of future therapies or serve as an adjunct to help treat metabolic perturbations.

Understanding the mechanisms responsible for anterior segment diabetic disease will uncover potential biomarkers in the aqueous humor that can be used to determine the health status of the anterior segment and the degree to which diabetes influences anterior segment physiology. These mechanisms may also provide insight towards potential therapeutic targets for slowing or even halting the progression of anterior segment diabetic disease.

## Supporting information

S1 Fig Gene ontology protein classes for differentially expressed proteins due to diabetic severity. (TIF)

S1 Table Complete metabolomic dataset. (XLSX)

S2 Table Diabetic medication comparison. (XLSX)

S3 Table Complete metabolomic 4-way pathway. (XLS)

S4 Table Complete proteomic dataset. (XLS)

S5 Table Global protein expression changes. (XLSX)

S6 Table Complete proteomic 4-way pathway. (XLS)

S7 Table Common metabolomic and proteomic. (XLS)
