# Supplementary figures and images for: Metabolic and proteomic indications of diabetes progression in human aqueous humor
Source: PLoS One. 2023 Jan 18;18(1):e0280491. doi: 10.1371/journal.pone.0280491 (PMC9847982; doi:10.1371/journal.pone.0280491)

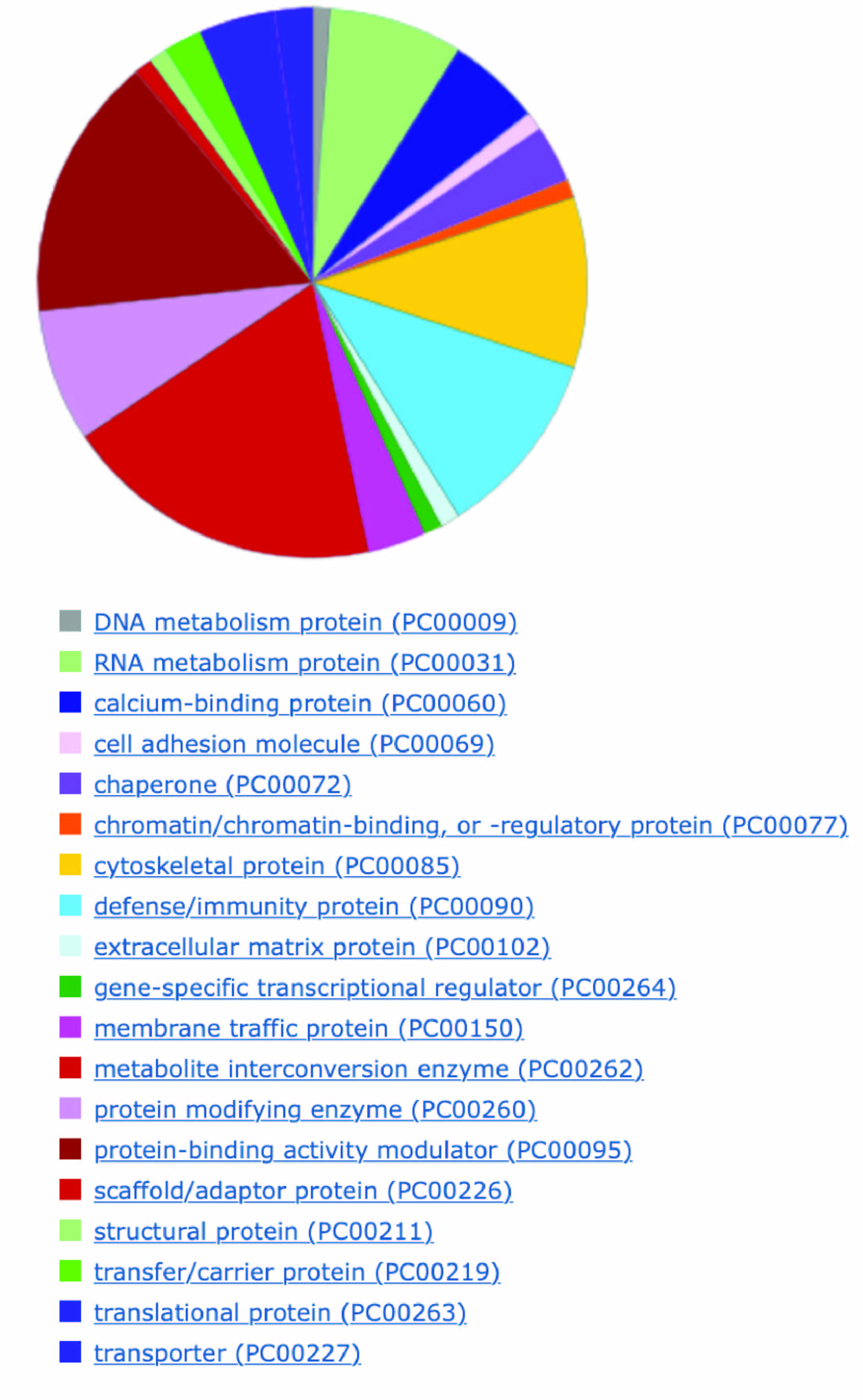

Supplement: S1 Fig — (TIF) [file pone.0280491.s001.tif]
